# A systematic review and network meta-analysis of single nucleotide polymorphisms associated with oral submucous fibrosis risk

**DOI:** 10.1097/MD.0000000000050594

**Published:** 2026-09-04

**Authors:** Chunxia Huang, Juan Liu, Bin Zeng, Lele Zhang, Zhihang Zhou, Xiaoshan Huang, Binsheng He, Meihua Bao

**Affiliations:** a Department of Oral Basic Science, School of Stomatology, Changsha Medical University, Changsha, China; b Department of Pharmacology, College of Pharmacy, Changsha Health Vocational College, Changsha, Hunan, China; c Department of Basic Medicine Science, Hunan Provincial Key Laboratory of the TCM Agricultural Biogenomics, Changsha Medical University, Changsha, China; d Department of Pharmacology, Hunan key Laboratory of the Research and Development of Novel Pharmaceutical Preparations, School of Pharmaceutical Science, Changsha Medical University, Changsha, China.

**Keywords:** network meta-analysis, oral submucous fibrosis, single nucleotide polymorphism

## Abstract

**Background:**

Oral submucous fibrosis (OSF) is a chronic and insidious oral disease characterized by hyalinization of the subepithelial connective tissue and progressive fibrosis of the oral submucosa. It is a precancerous condition of oral squamous cell carcinoma. Studies have demonstrated that single nucleotide polymorphisms (SNPs) are closely associated with susceptibility to OSF. This study aims to comprehensively evaluate the association between SNPs and OSF risk and to rank the strength of the association between different genetic models and OSF susceptibility.

**Methods:**

Literature related to OSF was comprehensively searched from PubMed, Web of Science, Embase, Cochrane Library, CNKI, and Wangfang databases up to July 2025. Full-text case-control studies with patients diagnosed with OSF were included. Quality assessment was performed to evaluate the risk of bias. RevMan 5.4, GeMTC 0.14.3, and STATA 17.0 were used for the pairwise and Bayesian network meta-analysis.

**Results:**

A total of 24 studies with 2545 cases and 3772 controls, covering 13 SNPs in 11 genes, were included in our meta-analysis. We found that CYP1A1 rs4646903:T>C, CYP1A1 rs1048943:A>G, GSTT1 null genotype, GSTM1 null genotype, and XRCC3 rs861539:C>T were associated with an increased risk of OSF, while MMP2 rs243865:C>T and MMP3 rs3025058: 5A>6A were associated with a decreased risk of OSF. Further Bayesian network meta-analysis indicated the top 5 genetic models with the highest association with OSF risk in network group 1 were the dominant model, homozygous model, allelic model, and recessive model of CYP1A1 rs1048943:A>G (ranked 1–4), and the heterozygous/dominant model of CYP1A1 rs4646903:T>C (both ranked 5). While the allelic models of XRCC3 rs861539:C>T and MMP3 rs3025058: 5A>6A ranked first for predicting OSF in group 2 and group 3, respectively.

**Conclusion:**

Some specific SNPs are significantly related to the risk of OSF. Among them, the dominant model of CYP1A1 rs1048943:A>G may be the most strongly associated genetic model with OSF risk. Future large-sample, well-designed studies with detailed genotype data are needed to validate the roles of these SNPs in OSF risk.

**Key Points**

1. Systematically analyzed the relations of SNPs to OSF risk.
2. Ranked the SNPs associated with OSF risk using network meta-analysis.
3. Identified the relative strengths of association between different genetic models and OSF risk.

## 1. Introduction

Oral submucous fibrosis (OSF) is a chronic and insidious oral disease characterized by hyalinization of the oral connective tissue and progressive fibrosis of the oral submucosa.^[1]^ The prevalence of OSF ranges from 2.28% to 8.62%, with a notably higher incidence observed among betel nut consumers in Asian countries. OSF is recognized as a precancerous condition of oral squamous cell carcinoma (OSCC), with a malignant transformation rate as high as 7% to 13%. The World Health Organization (WHO) has classified OSF as one of the oral potentially malignant disorders.^[2]^ Investigating the etiology and progression of malignant transformation in OSF is critical for both the treatment of OSF and the prevention of OSCC. The risk factors for the pathogenesis of OSF include prolonged exposure to betel nuts, intake of spicy foods, smoking, and alcohol consumption. In addition, nutritional deficiencies, immune regulation imbalance, genetic predisposition, and hemorheological abnormalities also contribute to the onset and progression of OSF.^[1,3]^

Studies have demonstrated that single nucleotide polymorphisms (SNPs) in different genes are associated with the susceptibility and clinical severity of different diseases.^[4-7]^ The reported SNPs associated with OSF include transforming growth factor-β1 (TGFB1),^[8]^ matrix metalloproteinase (MMP) family,^[9]^ glutathione S-transferases (such as GSTM1 and GSTT1),^[10]^ and the cytochrome P450 family (e.g., CYP1A1 and CYP2E1).^[11]^

Even though many clinical studies have explored the association between SNPs and OSF susceptibility, the findings across these studies are inconsistent. Furthermore, the relative strength of association between different SNPs and OSF risk remains unclear.^[12,13]^ Network meta-analysis (NMA) is a statistical methodology that integrates and weights data from both direct and indirect comparisons.^[14]^ It can be employed to compare multiple SNPs and provide a comprehensive evaluation of their relationship with a disease.

This study intends to employ both direct meta-analysis and Bayesian network meta-analysis to systematically and comprehensively evaluate the relationship between SNPs and OSF susceptibility, with the aim of identifying the relative strength of association between different SNPs and OSF risk.

## 2. Materials and methods

### 2.1. Literature search and data extraction

**Figure 1.**
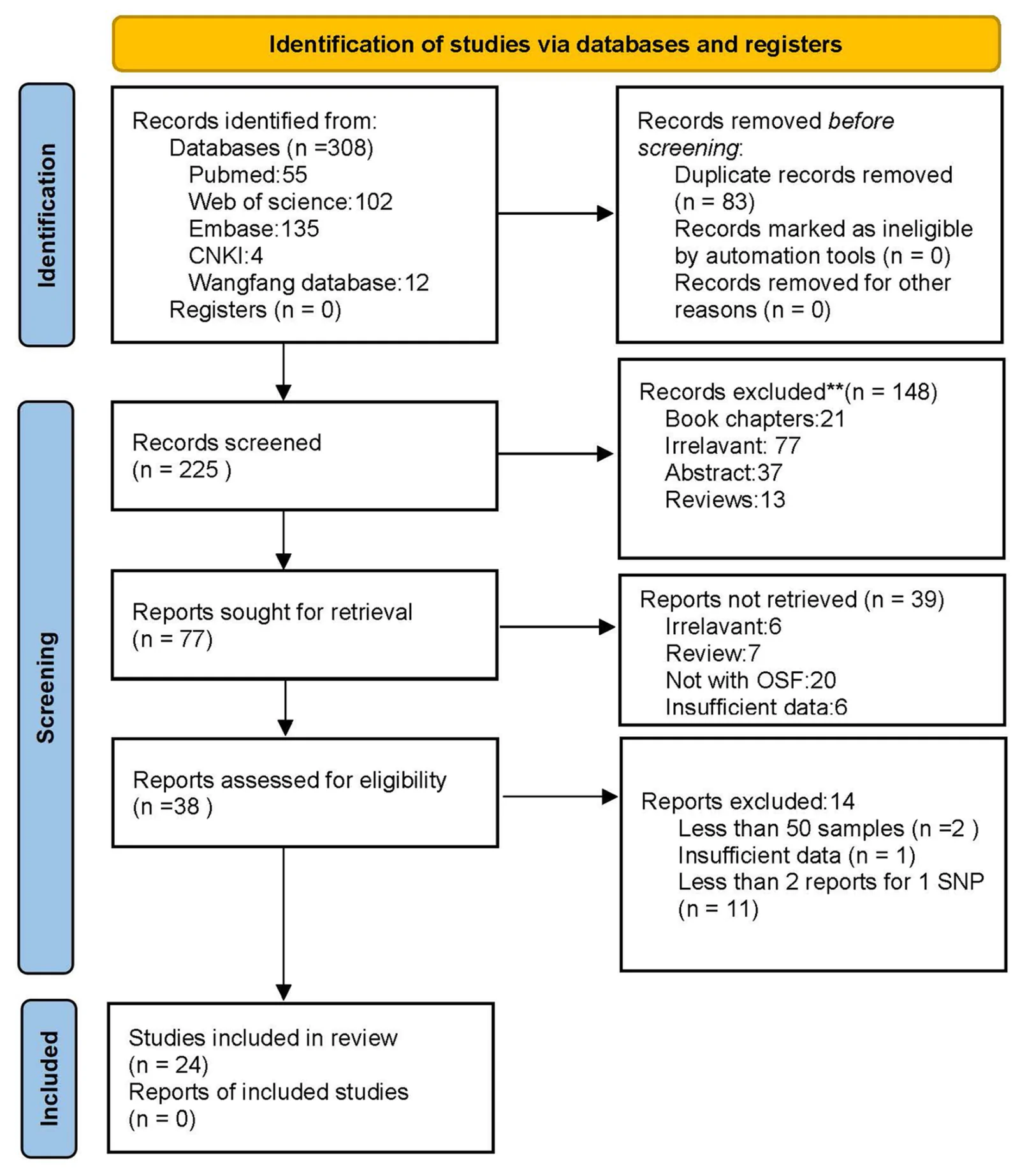
PRISMA flow diagram for the inclusion and exclusion of searched studies. CNKI = China National Knowledge Infrastructure.

We used terms such as “single nucleotide polymorphism,” “SNP,” “polymorphism,” “oral submucous fibrosis,” “OSF” to search PubMed, Web of Science, Embase, China National Knowledge Infrastructure (CNKI), and Wanfang databases for studies published up to July 2025 without any language restrictions. The study was designed and performed in accordance with the PRISMA guidelines (Fig. 1).

Inclusion criteria were: full-text case-control studies; patient diagnosed with oral submucous fibrosis; comparing gene SNP frequencies between OSF patients and healthy controls; with sufficient genotype information. The exclusion criteria were: non-full-text case-control studies; non-original research (e.g., reviews, case reports, conference abstracts), animal studies, or commentaries; patients diagnosed with precancerous lesions or potentially malignant disorder; non-English or Chinese articles; total sample size <10; <2 reports for a single SNP; demonstrated a high risk of bias (assessed as a score < 7 on the quality assessment tool); reported contradictory or implausible results.

Data extraction was performed independently by 2 reviewers (Huang C and Zhou Z). Any disagreements were resolved by a third independent reviewer (He B) was involved to reach consensus. We extracted data including author names, year of publication, country, sample size, gender, Hardy–Weinberg equilibrium (HWE) values, and genotyping methods. Data were methodically evaluated by 2 independent reviewers (Zeng B and Zhang L) according to the guidelines of the STREGA statement.^[15]^ The third reviewer (Liu J) was involved in resolving any issues between the 2 reviewers. The corresponding authors were contacted if any data were missing, insufficient, or vague. However, if relevant data was not obtained, those studies were excluded.

### 2.2. Quality evaluation for included studies

The quality of each involved study was evaluated according to previous studies.^[16,17]^ Briefly, the representative of cases (2 points), representative of controls (2 points), ascertainment of diseases (2 points), case-control matching (2 points), genotyping examination (1 point), HWE (1 point), and total sample size (3 points) were taken into account and given a corresponding score. Total scores ranged from 0 to 13. Studies were categorized as high quality (low risk of bias) with a score >10, medium quality (intermediate or unknown risk of bias) with a score of 7 to 10, or poor quality (high risk of bias) with a score < 7. The quality evaluation was conducted by 2 authors independently. If any discrepancy was found, a consensus was reached.

### 2.3. Pairwise meta-analysis and false positive report probability (FPRP)

Pairwise meta-analysis was conducted using RevMan 5.4 software (The Cochrane Collaboration, 2020). Pooled odds ratios (ORs) with 95% confidence intervals (CIs) were calculated using either fixed-effects (FE) or random-effects (RE) model, depending on the heterogeneity of the genetic models for each SNP. Heterogeneity between studies was evaluated using the *I^2^* statistic and *P* value. A FE model was used for studies with low heterogeneity (*I^2^* < 50% and *P* > .1). Otherwise, RE model was used.

Given the small number of included studies (n < 10), leave‑one‑out sensitivity analysis was not performed. Instead, the sensitivity analysis was conducted by re‑analyzing the data using both FE and RE models to examine the consistency of the pooled ORs and 95% CI. For studies deviating from HWE, we recalculated the pooled effect size and 95% CI after excluding studies that deviated from HWE to evaluate their impact on the overall results.

Publication bias was assessed via visual inspection of funnel plots (plotting effect size against standard error). Formal statistical tests for funnel plot asymmetry (Egger regression test and Begg rank correlation test) were not performed, as the number of included studies was fewer than 10, which renders such tests under-powered and unreliable for detecting publication bias.

FPRP was calculated by setting a prior probability of 0.01 and a statistical power of 1.5. Genetic models with FPRP value < 0.2 were considered credible.

### 2.4. Network meta-analysis (NMA)

For SNPs with significant association with OSF susceptibility, we conducted NMA. We used GeMTC software (version 0.14.3; DrugIS project, University Medical Center Groningen) and STATA software (version 17.0; StataCorp LLC) for the NMA. It was based on the Bayesian framework and Markov Chain Monte Carlo (MCMC) theory to generate a network relationship diagram for SNPs related to OSF risk. The 4 parallel MCMC simulations underwent a burn-in phase of 20,000 simulations and then an additional iteration phase of 50,000 simulations. The outcomes were evaluated by OR and 95% CI under a random-effects model. A consistency model was applied if the 95% CI of log (OR) included 0. Otherwise, an inconsistency model was used. The potential scale reduction factor (PSRF) was used to determine convergence. The model was considered convergent if the value of PSRF was close to 1.0. This Bayesian method was used to rank each genetic model according to the probability of OSF risk, and the corresponding rank probability map was automatically generated.

## 3. Results

### 3.1. Literature search results

The literature search initially identified 308 studies from PubMed, Web of Science, Embase, CNKI, and Wanfang databases. The search ended on July 10, 2025. As shown in Figure 1, 225 records were obtained after duplicates were removed. Among them, 21 were book chapters, 83 were irrelevant, 37 were abstracts, 20 were reviews or meta-analysis, 20 focus on other diseases, and 6 lacked sufficient data for analysis. After removing these articles, 38 articles were screened out for further assessment.

Of these 38 articles, the study by Zade included only 10 samples,^[18]^ the study by Thorawat A was of low quality,^[19]^ the study by Yaming lacked sufficient data,^[20]^ and 11 studies investigated SNPs with <2 reports^[21–31]^; thus, they were excluded. Finally, 24 studies with 2545 cases and 3772 controls were included in our analysis. These studies analyzed 11 genes and 13 SNPs, including lysyl oxidase (LOX) rs1800449:G>A,^[2,8,32]^ cytochrome P450 Family 1 Subfamily A Member 1 (CYP1A1) rs4646903:T>C^[10,11]^ and rs1048943:A>G,^[10,11]^ glutathione S-transferase T1 (GSTT1) null genotype,^[10,33-35]^ glutathione S-transferase M1 (GSTM1) null genotype,^[10,33-35]^ matrix metalloproteinases-1 (MMP1) rs1799750:1G>2G,^[36,37]^ matrix metalloproteinases-2 (MMP2) rs243865:C>T,^[9,38]^ matrix metalloproteinases-3 (MMP3) rs 3025058:5A>6A,^[39,40]^ matrix metalloproteinases-9 (MMP9) rs3918242: C>T^[9,41,42]^ and rs17576:G>A,^[42,43]^ x-ray repair cross-complementing 3 (XRCC3) rs rs861539:C>T,^[44,45]^ TP53 rs1042522:G>C,^[46–49]^ and transforming growth factor-β1 (TGFB1) rs1800469:T>C^[8,50]^ (PRISMA flow diagram, Tables 1 and S1, Supplemental Digital Content 1).

**Table 1:** Included SNPs and their corresponding literatures.

	GENE	SNP
1	Lysyl oxidase (LOX)	rs1800449:G>A^[2,8,32]^
2	Cytochrome P450 Family 1 Subfamily A Member 1 (CYP1A1)	rs4646903:T>C,^[10,11]^ rs1048943:A>G^[10,11]^
3	Glutathione S-transferase T1 (GSTT1)	Null genotype^[10,33-35]^
4	Glutathione S-transferase M1 (GSTM1)	Null genotype^[10,33-35]^
5	Matrix metalloproteinases-1 (MMP1)	rs1799750:1G>2G^[36,37]^
6	Matrix metalloproteinases-2 (MMP2)	rs243865:C>T^[9,38]^
7	Matrix metalloproteinases-3 (MMP3)	rs 3025058:5A>6A^[39,40]^
8	Matrix metalloproteinases-9 (MMP9)	rs3918242: C>T,^[9,41,42]^ rs17576:G>A^[42,43]^
9	X-ray repair cross-complementing 3 (XRCC3)	rs rs861539:C>T^[44,45]^
10	TP53	rs1042522:G>C^[46-49]^
11	Transforming growth factor-β1 (TGFB1)	rs1800469:T>C^[8,50]^

CYP1A1 = Cytochrome P450 Family 1 Subfamily A Member 1, GSTM1 = glutathione S-transferase M1, GSTT1 = glutathione S-transferase T1, LOX = lysyl oxidase, MMP1 = matrix metalloproteinases-1, MMP2 = matrix metalloproteinases-2, MMP3 = matrix metalloproteinases-3, MMP9 = matrix metalloproteinases-9, SNP = single nucleotide polymorphism, TGFB1 = transforming growth factor-β1, XRCC3 = x-ray repair cross-complementing 3.

### 3.2. Quality evaluation

The quality assessment of included studies is presented in Table 2. Most of the studies were of high or medium quality (low or intermediate risk of bias). Only one study^[19]^was of low quality (high risk of bias); thus, it was excluded from our analysis. Five studies deviated from HWE in the control group; we performed sensitivity analyses by excluding HWE-deviating studies to evaluate their influence on pooled estimates. Most of the studies had good representation of cases or controls, adequate case-control matching, reliable genotyping examination, and compliance with HWE. The sample size of most studies ranged from 200 to 500.

**Table 2:** Quality assessment of involved studies.

Author, year	A	B	C	D	E	F	G	Total	Author	A	B	C	D	E	F	G	Total
Shieh TM, 2009^[32]^	2	2	1	2	1	1	1	10	Tu HF, 2008^[47]^	2	1	2	2	1	1	1	10
Chaudhary AK, 2011^[9]^	2	2	2	2	1	0	2	11	Majumdar PK, 2013^[43]^	2	1	1	2	1	1	0	8
Mannan A, 2024^[35]^	2	1	2	2	1	-	1	9	Hallikeri K, 2018^[48]^	2	1	1	1	1	1	0	7
Mukherjee S, 2011^[44]^	2	2	2	2	1	1	0	10	Lin YC, 2008^[46]^	2	2	2	2	1	1	1	11
Chaudhuri SR, 2013^[11]^	2	2	1	2	1	1	1	10	Ghosh T, 2012^[10]^	2	2	1	2	1	1	1	10
Mukherjee S, 2021^[2]^	2	2	2	2	1	0	1	10	Agrawal D, 2010^[33]^	2	1	2	2	1	-	1	9
Tu HF, 2007^[41]^	2	1	1	2	1	1	1	9	Katarkar A, 2018^[42]^	2	1	1	2	1	1	1	9
Chaudhary AK(1), 2010^[37]^	2	2	2	2	1	1	2	12	Yaming P, 2016^[20]^	2	2	1	2	1	0	0	8
Madhulatha G, 2018^[34]^	2	1	1	2	1	-	0	7	Chaudhary AK (2), 2010^[40]^	2	2	1	2	1	1	1	10
Chiu CJ, 2002^[8]^	2	2	2	0	1	0	1	8	Tu HF, 2006^[39]^	2	1	1	2	1	1	0	8
Lin SC(2), 2004^[38]^	2	2	1	2	1	1	1	10	Lin SC(1), 2004^[36]^	2	2	2	2	1	1	1	11
Rajendran R, 2010^[50]^	2	1	2	0	1	1	0	7	Nigam K, 2023^[45]^	2	1	1	2	1	0	1	8

A: representative of cases (2 points); B: representative of controls (2 points); C: ascertainment of diseases (2 points); D: case-control matching (2 points); E: genotyping examination (1 point); F: HWE (1 point); G: total sample size (3 points). Columns A‑G denote study quality ratings: green indicates full scores, orange indicates partial scores, and red indicates zero scores. In the Total score column, green indicates high-quality studies and orange indicates moderate‑quality studies.

HWE = Hardy-Weinberg equilibrium.

### 3.3. Pairwise meta-analysis and FPRP

As shown in Table 3, a total of 13 SNPs in 11 genes were evaluated for their associations with OSF risk. Five genetic models for each SNP were used for the analysis.

**Table 3:** Pairwise meta-analysis of the selected SNPs in association with risk for OSF.

Genetic model	Sample size	Studies	Heterogeneity		Model	OR (95%CI)	FPRP of 0.01 prior probability
	Case/control	N	*I^2^* (%)	*P*			
LOX rs1800449:G>A	282/413	3					
AA vs GG			0	.37	Fixed	1.39 [0.68, 2.88]	0.961
AG vs GG			67	.28	Random	1.41 [0.76, 2.61]	0.948
AA vs AG + GG			0	.59	Fixed	1.22 [0.59, 2.53]	0.974
AA + AG vs GG			70	.29	Random	1.40 [0.75, 2.58]	0.950
A vs G			69	.30	Random	1.30 [0.79, 2.16]	0.952
CYP1A1 rs4646903:T>C	177/350	2					
CC vs TT			0	<.00001	Fixed	4.35 [2.41, 7.86]	<0.0006
CT vs TT			54	.03	Random	2.08 [1.06, 4.08]	0.664
CC vs CT + TT			35	<.00001	Fixed	3.27 [2.07, 5.17]	<0.0006
CC + CT vs TT			0	<.00001	Fixed	2.65 [1.72, 4.09]	<0.0006
C vs T			0	<.00001	Fixed	2.26 [1.73, 2.97]	<0.0006
CYP1A1 rs1048943:A>G	272/430	3					
GG vs AA			83	.0005	Random	5.87 [2.16, 15.97]	0.032
GA vs AA			N.A	.0003	Fixed	6.19 [2.32, 16.50]	0.019
GG vs GA + AA			74	<.0001	Random	4.23 [2.14, 8.37]	0.026
GG + GA vs AA			81	.0004	Random	5.55 [2.16, 14.25]	<0.006
G vs A			87	<.0001	Random	4.52 [2.21, 9.26]	<0.006
GSTT1 null genotype	325/462	4					
Null genotype (-/-) vs Active genotype (+/+ and +/-)			0	<.00001	Fixed	2.47 [1.69, 3.59]	<0.0006
GSTM1 null genotype	325/462	4					
Null genotype (-/-) vs Active genotype (+/+ and +/-)			20	<.0001	Fixed	1.81 [1.35, 2.43]	<0.006
MMP1 rs1799750:1G > 2G	470/573	2					
2G2G vs 1G1G			69	.68	Random	1.22 [0.47, 3.18]	0.978
2G1G vs 1G1G			0	.77	Fixed	0.96 [0.74, 1.25]	0.981
2G2G vs 2G1G + 1G1G			72	.66	Random	1.24 [0.48, 3.18]	0.978
2G2G + 2G1G vs 1G1G			0	.73	Random	1.04 [0.81, 1.34]	0.979
2G vs 1G			46	.25	Fixed	1.12 [0.93, 1.35]	0.943
MMP2 rs243865:C>T	470/569	2					
TT vs CC			12	.35	Fixed	0.74 [0.40, 1.39]	0.959
TC vs CC			11	.96	Fixed	0.98 [0.51, 1.91]	0.984
TT vs TC + CC			13	.48	Fixed	0.80 [0.43, 1.49]	0.969
TT + TC vs CC			0	.03	Fixed	0.73 [0.55, 0.97]	0.664
T vs C			0	.03	Fixed	0.76 [0.59, 0.97]	0.664
MMP3 rs3025058:5A > 6A	171/224	2					
6A6A vs 5A5A			0	.35	Fixed	0.49 [0.11, 2.21]	0.958
6A5A vs 5A5A			0	.77	Fixed	1.26 [0.26, 6.15]	0.981
6A6A vs 6A5A + 5A5A			0	.0004	Fixed	0.39 [0.23, 0.66]	0.026
6A6A + 6A5A vs 5A5A			0	.47	Fixed	0.57 [0.13, 2.61]	0.969
6A vs 5A			0	.0005	Fixed	0.44 [0.28, 0.70]	0.032
MMP9 rs3918242:C>T	674/809	3					
TT vs CC			57	.57	Random	1.35 [0.48, 3.77]	0.974
TC vs CC			0	.57	Fixed	0.93 [0.72, 1.19]	0.974
TT vs TC + CC			62	.62	Random	1.32 [0.44, 4.00]	0.976
TT + TC vs CC			0	.55	Fixed	1.07 [0.85, 1.36]	0.973
T vs C			0	.25	Fixed	1.13 [0.92, 1.38]	0.943
MMP9 rs17576:G>A	229/236	2					
AA vs GG			69	.75	Random	1.23 [0.34, 4.41]	0.980
AG vs GG			51	.75	Random	1.12 [0.54, 2.35]	0.980
AA vs AG + GG			49	.21	Fixed	1.43 [0.81, 2.52]	0.933
AA + AG vs GG			68	.81	Random	1.11 [0.46, 2.69]	0.982
G vs A			76	.76	Random	1.12 [0.55, 2.28]	0.980
XRCC3 rs861539:C>T	165/391	2					
TT vs CC			43	<.0001	Fixed	6.86 [2.63, 17.88]	<0.006
TC vs CC			70	.45	Random	1.35 [0.62, 2.93]	0.967
TT vs TC + CC			62	.02	Random	6.29 [1.35, 29.34]	0.569
TT + TC vs CC			56	.13	Random	1.60 [0.87, 2.93]	0.896
T vs C			5	.0008	Fixed	1.71 [1.25, 2.33]	0.050
TP53 rs1042522:G>C	135/371	3					
CC vs GG			0	.75	Fixed	1.10 [0.61, 2.01]	0.980
GC vs GG			0	.44	Fixed	0.83 [0.52, 1.32]	0.967
CC vs CG + GG			25	.86	Fixed	1.05 [0.61, 1.80]	0.983
CC + CG vs GG			0	.69	Random	0.92 [0.60, 1.41]	0.978
C vs G			9	.93	Fixed	1.01 [0.75, 1.36]	0.984
TGFB1 rs1800469:T > C	215/333	2					
CC vs TT			-	.55	Fixed	1.19 [0.68, 2.07]	0.973
CT vs TT			8	.32	Fixed	0.80 [0.52, 1.23]	0.955
CC vs CT + TT			-	.13	Fixed	1.44 [0.90, 2.31]	0.896
CC + CT vs TT			0	.61	Fixed	0.90 [0.60, 1.35]	0.97
C vs T			0	.61	Fixed	0.90 [0.60, 1.35]	0.976

CI = confidence interval, CYP1A1 = Cytochrome P450 Family 1 Subfamily A Member 1, FPRP = false positive report probability, GSTM1 = glutathione S-transferase M1, GSTT1 = glutathione S-transferase T1, LOX = lysyl oxidase, MMP1 = matrix metalloproteinases-1, MMP2 = matrix metalloproteinases-2, MMP3 = matrix metalloproteinases-3, MMP9 = matrix metalloproteinases-9, OR = odds ratio, OSF = oral submucous fibrosis, SNP = single nucleotide polymorphism, TGFB1 = transforming growth factor-β1, XRCC3 = x-ray repair cross-complementing 3.

For CYP1A1 rs4646903:T>C, all 5 genetic models were related to an increased risk of OSF (CC vs TT, OR = 4.35, 95% CI: 2.41–7.86, *P* < .00001; CT vs TT, OR = 2.08, 95% CI: 1.06–4.08, *P* = .03; CC vs CT + TT, OR = 3.27, 95% CI: 2.07–5.17, *P* < .00001; CC + CT vs TT, OR = 2.65, 95% CI: 1.72–4.09, *P* < .00001; C vs T, OR = 2.26, 95% CI: 1.73–2.97, *P* < .00001). All of them had FPRP < 0.2, except the heterozygous model (TT vs TC, FPRP = 0.664).

For CYP1A1 rs1048943:A>G, all 5 genetic models were associated with an increased risk of OSF (GG vs AA, OR = 5.87, 95% CI: 2.16–15.97, *P* = .0005; GA vs AA, OR = 6.19, 95% CI: 2.32–16.50, *P* = .0003; GG vs GA + AA, OR = 4.23, 95% CI: 2.14–8.37, *P* < .0001; GG + GA vs AA, OR = 5.55, 95% CI: 2.16–14.25, *P* = .0004; G vs A, OR = 4.52, 95% CI: 2.21–9.26, *P* < .0001). All of these genetic models had FPRP < 0.2.

For GSTT1 and GSTM1 homozygous deletions, the null (−/−) genotype was associated with an increased risk of OSF compared to the active (+/+ and +/−) genotype (GSTT1, null vs active, OR = 2.47, 95% CI: 1.69–3.59, *P* < .00001; GSTM1, null vs active, OR = 1.81, 95% CI: 1.35–2.43, *P* < .0001). Both of them had FPRP < 0.0006.

For MMP2 rs243865:C>T, the dominant model and allelic model were associated with a decreased risk of OSF (TT + TC vs CC, OR = 0.73, 95% CI: 0.55–0.97, *P* = .03; T vs C, OR = 0.76, 95% CI: 0.59–0.97, *P* = .03). However, the FPRP values of these 2 models were above 0.2.

For MMP3 rs3025058: 5A>6A, the recessive and allelic models were associated with a decreased risk of OSF (6A6A vs 6A5A + 5A5A, OR = 0.39, 95% CI: 0.23–0.66, *P* = .0004; 6A vs 5A, OR = 0.44, 95% CI: 0.28–0.70, *P* = .0005), with both FPRP values below 0.2.

For XRCC3 rs861539:C>T, homozygous, recessive, and allelic models were associated with an increased risk of OSF (TT vs CC, OR = 6.86, 95% CI: 2.63–17.88, *P* < .0001, FPRP < 0.006; TT vs TC + CC, OR = 6.29, 95% CI: 1.35–29.34, *P* = .02, FPRP = 0.569; T vs C, OR = 1.71, 95% CI: 1.25–2.33, *P* = .0008, FPRP = 0.050).

Overall, the pairwise meta-analysis revealed significant associations between multiple genetic polymorphisms and OSF risk: CYP1A1 rs4646903:T>C, CYP1A1 rs1048943:A>G, GSTT1 null, GSTM1 null, and XRCC3 rs861539:C>T were associated with an increased risk of OSF, with most significant models supported by low FPRP values indicating robust credibility. In contrast, MMP3 rs3025058:5A>6A was associated with a decreased OSF risk in credibly significant models, whereas the protective associations observed for MMP2 rs243865:C>T lacked sufficient credibility due to high FPRP values.

### 3.4. Sensitivity analysis and publication bias analysis

To assess the robustness and reliability of our meta-analytic estimates, we conducted sensitivity analyses based on different effect models, the exclusion of HWE-deviating studies, and the evaluation of publication bias. The sensitivity analysis indicated that no obvious difference was found between the RE and FE models for pooled ORs and 95% CI for variant genetic models in LOX rs1800449:G>A, CYP1A1 rs4646903:T>C, CYP1A1 rs1048943:A>G, GSTT1 null genotype, GSTM1 null genotype, MMP2 rs243865:C>T, MMP3 rs3025058:5A>6A, MMP9 rs3918242:C>T, MMP9 rs17576:G>A, TP53 rs1042522:G>C, and TGFB1 rs1800469:T>C. Only slightly wider 95% CIs were found in the RE model compared with the FE model for MMP1 rs1799750:1G>2G homozygous and recessive models, MMP9 rs3918242: C>T recessive model, MMP9 rs17576:G>A homozygous model, and XRCC3 rs861539:C>T homozygous and recessive models, reflecting the inclusion of between-study heterogeneity in the random-effects model (Table S2, Supplemental Digital Content 2). Despite the minor difference in precision, the direction and statistical significance of the pooled effect remained unchanged across both models, supporting the overall robustness of the findings.

Among the included 24 studies, 5 studies deviated from HWE (controls: *P* < .05, Table 2). After excluding the study by Mukherjee^[2]^ for LOX rs1800449:G > A, the pooled OR and 95% CI, as well as the heterogeneity (*I^2^*), were highly consistent with the primary analysis (all studies included), indicating that the HWE-deviant study did not materially influence the overall findings (Table S2, Supplemental Digital Content 2). When excluding the study by Yaming^[20]^ for CYP1A1 rs1048943:A>G, the heterogeneity decreased from 83% to 0% in the homozygous model and from 81% to 0% in the dominant model, indicating that it might be the source of heterogeneity in these models (Table S2, Supplemental Digital Content 2). For MMP2 rs243865:C>T, XRCC3 rs861539:C>T, and TGFB1 rs1800469:T>C, one of the 2 included studies deviated from HWE in the control group. Owing to the small number of included studies (n = 2), sensitivity analysis excluding this study was not possible, as the exclusion of any single study would leave only 1 study, precluding meaningful pooled analysis.

We performed funnel plots to evaluate publication bias. No obvious publication bias was observed for the included SNPs (data not shown).

Collectively, these sensitivity analyses confirmed that our main results were stable and reliable, largely unaffected by effect model choice, HWE deviations, or publication bias.

### 3.5. Network meta-analysis (NMA)

**Figure 2.**
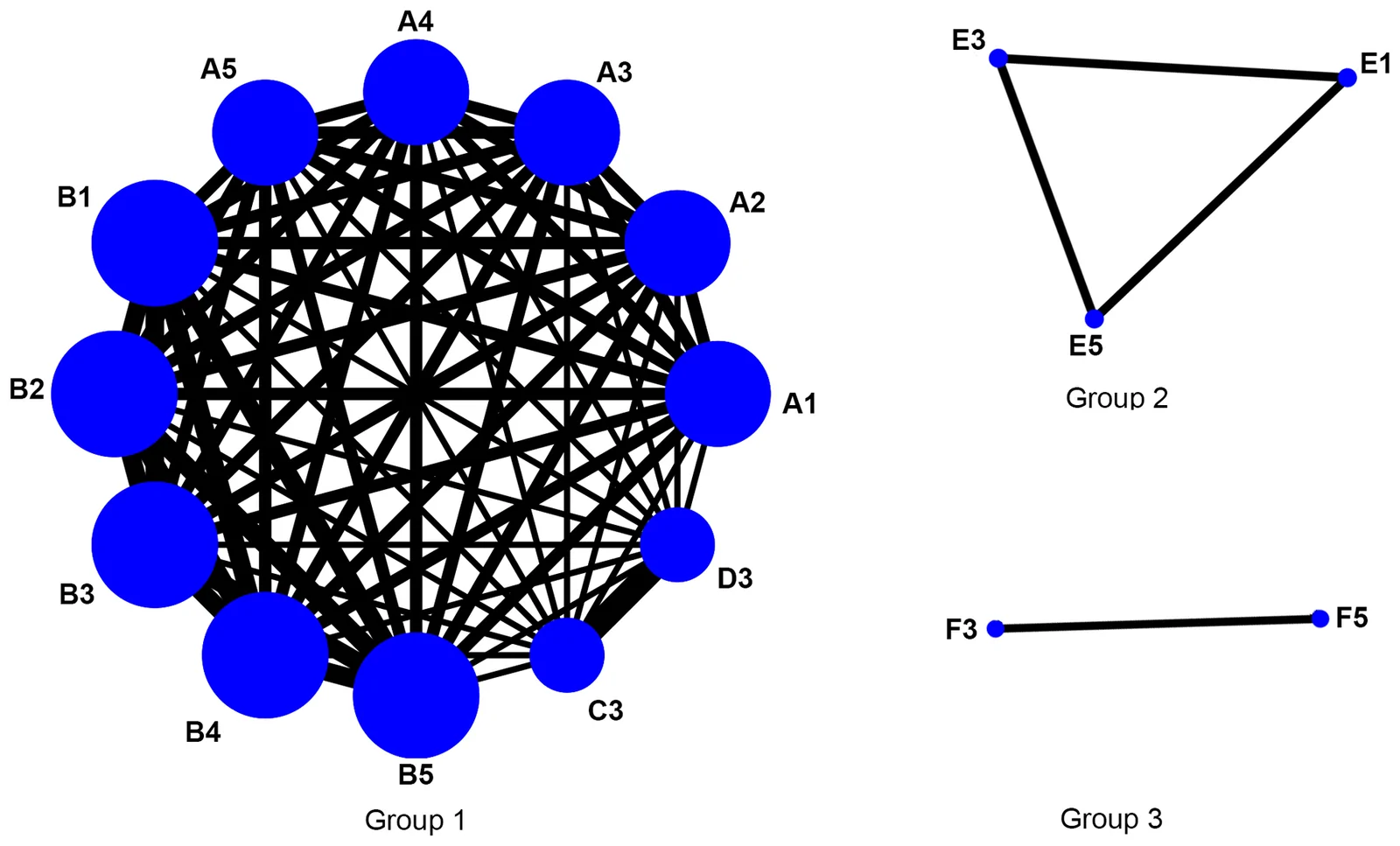
Network meta-analysis of genetic models for OSF risk-associated SNPs. The figure presents the network relationships for the (1) homozygous, (2) heterozygous, (3) recessive, (4) dominant, and (5) allelic genetic models of the following SNPs: (A) CYP1A1 rs4646903:T>C; (B) CYP1A1 rs1048943:A>G; (C) GSTT1 null genotype; (D) GSTM1 null genotype; (E) XRCC3 rs861539:C>T; (F) MMP3 rs3025058: 5A>6A. CYP1A1 = Cytochrome P450 Family 1 Subfamily A Member 1, GSTM1 = glutathione S-transferase M1, GSTT1 = glutathione S-transferase T1, MMP3 = matrix metalloproteinases-3, OSF = oral submucous fibrosis, SNP = single nucleotide polymorphism, XRCC3 = x-ray repair cross-complementing 3.

Following the pairwise meta-analysis, we further performed NMA to enable comparative evaluations across multiple genetic variants simultaneously. The NMA was conducted for SNPs significantly associated with OSF in pairwise meta-analysis. A total of 6 SNPs were involved in the NMA. The network showed 3 distinct groups (Fig. 2). Network group 1 consisted of various genetic models of CYP1A1 rs4646903:T>C, CYP1A1 rs1048943:A>G, GSTT1 null genotype, and GSTM1 null genotype. Network group 2 and 3 included various genetic models of XRCC3 rs861539:C>T or MMP3 rs3025058: 5A>6A, respectively.

### 3.6. Rank probabilities of different genetic models

**Figure 3.**
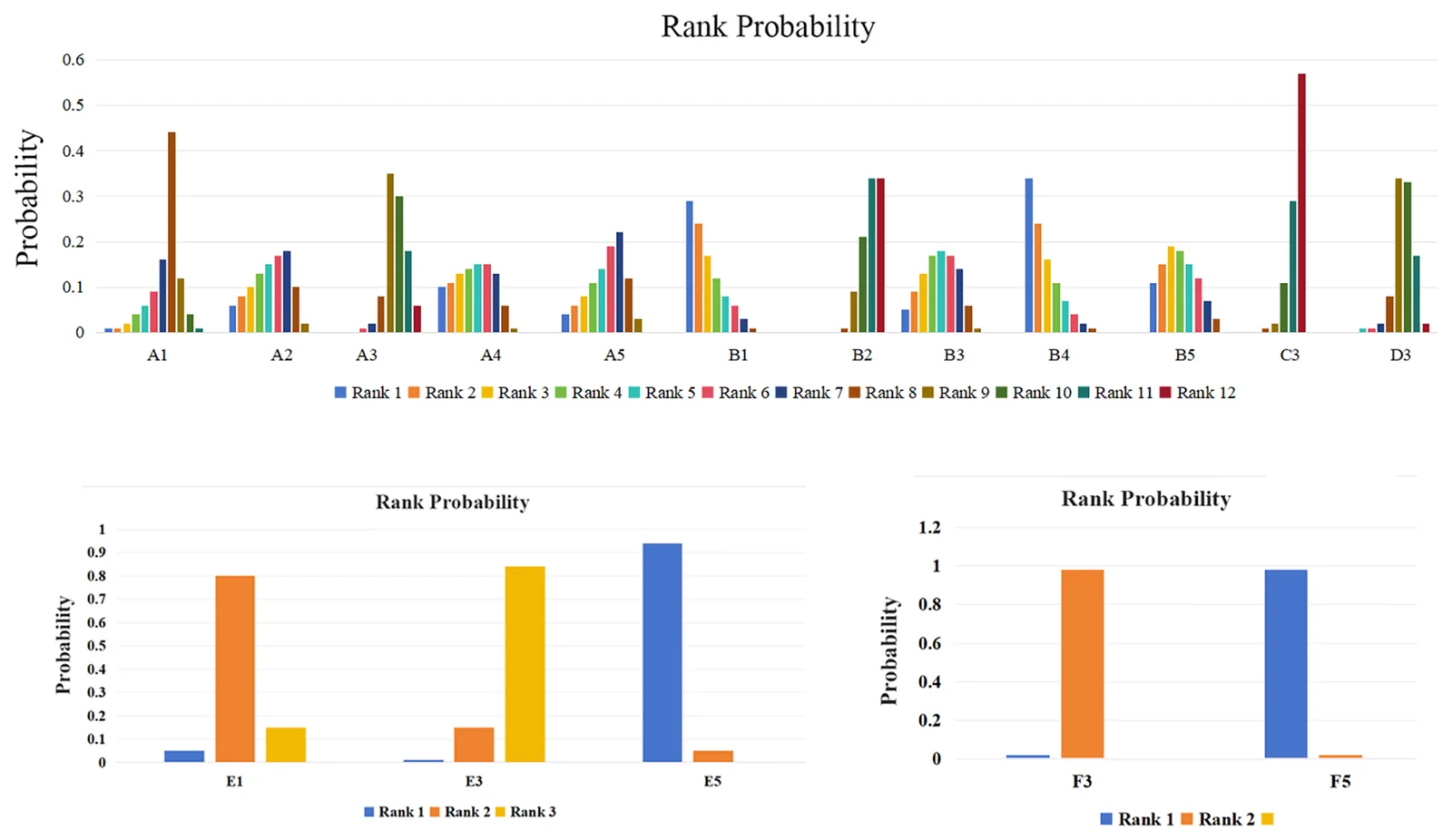
Rank probabilities of the 5 genetic models for OSF-associated SNPs, derived from network meta-analysis. Rank probabilities represent the posterior likelihood that each genetic model achieves a given rank (from best to worst) based on network meta-analysis results. The figures show the ranking probabilities for 5 genetic models: (1) homozygous, (2) heterozygous, (3) recessive, (4) dominant, and (5) allelic genetic models. Panels correspond to the following SNPs: (A) CYP1A1 rs4646903:T>C; (B) CYP1A1 rs1048943:A>G; (C) GSTT1 null genotype; (D) GSTM1 null genotype; (E) XRCC3 rs861539:C>T; (F) MMP3 rs3025058: 5A>6A. CYP1A1 = Cytochrome P450 Family 1 Subfamily A Member 1, GSTM1 = glutathione S-transferase M1, GSTT1 = glutathione S-transferase T1, MMP3 = matrix metalloproteinases-3, OSF = oral submucous fibrosis, SNP = single nucleotide polymorphism, XRCC3 = x-ray repair cross-complementing 3.

To further compare the relative strength of associations and evaluate evidence consistency across genetic models, we performed ranking probability analysis and inconsistency testing. As shown in Figure 3 and Tables S3–S5, Supplemental Digital Content 3–5, we calculated the possible ranking of the OSF-associated SNPs using GeMTC software. Our results identified the top 5 genetic models most strongly associated with OSF risk in group 1. The dominant model of CYP1A1 rs1048943:A>G ranked first, followed by its homozygous model (rank 2), allelic model (rank 3), the recessive model of CYP1A1 rs1048943:A>G (rank 4), and the heterozygous/dominant model of CYP1A1 rs4646903:T>C (both rank 5; Fig. 3). For group 2, the allelic model of XRCC3 rs861539:C>T ranked 1, followed by its homozygous model (rank 2) and the recessive model (rank 3). For group 3, the allelic model of MMP3 rs3025058:5A>6A ranked 1, followed by its recessive model (rank 2).

To verify the assumptions of transitivity and consistency in NMA, we supplemented a systematic evaluation with respect to key effect modifiers, including ethnicity, source of controls, age, gender distribution, and betel quid chewing prevalence. We confirmed that included studies were generally comparable across these key characteristics, supporting the transitivity assumption. Consistency was assessed by comparing agreement between direct and indirect evidence in GeMTC software.

Since indirect comparisons were only feasible within network group 1, inconsistency assessment was restricted to this subgroup. All PSRF values in group 1 were close to 1.0, supporting satisfactory convergence of the model results. Subsequent node-split analysis in GeMTC further evaluated the consistency between direct and indirect evidence for each comparison within this subgroup. The results indicated no inconsistency between the direct and indirect evidence in group 1 (all *P* > .05, Table 4). The strongest negative association in this subgroup was the comparison of dominant model of CYP1A1 rs1048943:A > G and the recessive model GSTT1 null genotype (standardized mean differences [SMD] of network OR = −3.37, 95% CI: −5.16 to −1.68). This was followed by the comparison between CYP1A1 rs1048943:A > G (homozygous model) vs. GSTT1 null genotype (recessive model; SMD of network OR = −3.37, 95% CI: −5.16 to −1.68), and CYP1A1 rs1048943:A > G (allelic model) vs. GSTT1 null genotype (dominant model; SMD of network OR = −3.01, 95% CI: −4.79 to −1.29).

**Table 4:** The direct and indirect evidence of each comparison in subgroup 1.

Name	Direct effect	Indirect effect	Overall	*P*-value
A1 vs C3	−2.23 (−4.81, 0.36)	−1.94 (−4.69, 0.77)	−1.98 (−3.94, −0.10)	.87
A1 vs D3	−1.30 (−3.91, 1.32)	−1.09 (−3.89, 1.65)	−1.05 (−2.99, 0.85)	.91
A2 vs C3	−2.20 (−4.80, 0.48)	−2.70 (−5.47, 0.03)	−2.70 (−4.63, −0.85)	.78
A2 vs D3	−1.21 (−3.76, 1.36)	−1.82 (−4.62, 0.89)	−1.77 (−3.65, 0.13)	.72
A3 vs C3	−0.95 (−3.53, 1.61)	−0.90 (−3.61, 1.84)	−0.93 (−2.75, 0.93)	.98
A3 vs D3	−0.01 (−2.63, 2.63)	−0.04 (−2.93, 2.74)	0.01 (−1.84, 1.87)	.99
A4 vs C3	−2.90 (−5.50, −0.30)	−2.83 (−5.59, −0.17)	−2.87 (−4.80, −1.01)	.97
A4 vs D3	−1.94 (−4.55, 0.69)	−1.97 (−4.64, 0.75)	−1.92 (−3.85, −0.06)	1
A5 vs C3	−1.79 (−4.35, 0.74)	−2.60 (−5.26, 0.12)	−2.60 (−4.48, −0.83)	.64
A5 vs D3	−0.85 (−3.44, 1.73)	−1.71 (−4.45, 0.95)	−1.66 (−3.52, 0.14)	.62
B1 vs C3	−3.51 (−6.29, −0.94)	−3.28 (−6.01, −0.62)	−3.31 (−5.13, −1.57)	.9
B1 vs D3	−2.61 (−5.37, 0.08)	−2.44 (−5.20, 0.19)	−2.38 (−4.18, −0.67)	.93
B2 vs C3	−1.81 (−4.59, 0.72)	−0.21 (−2.74, 2.85)	−0.30 (−2.07, 1.84)	.34
B2 vs D3	−0.88 (−3.80, 1.74)	0.64 (−2.00, 3.61)	0.63 (−1.12, 2.77)	.38
B3 vs C3	−2.11 (−4.70, 0.45)	−2.80 (−5.53, −0.18)	−2.81 (−4.59, −1.08)	.69
B3 vs D3	−1.16 (−3.81, 1.55)	−1.90 (−4.54, 0.72)	−1.88 (−3.67, −0.13)	.67
B4 vs C3	−3.77 (−6.42, −1.12)	−3.37 (−6.04, −0.75)	−3.37 (−5.16, −1.68)	.81
B4 vs D3	−2.78 (−5.49, −0.19)	−2.49 (−5.18, 0.16)	−2.43 (−4.21, −0.73)	.86
B5 vs C3	−2.74 (−5.23, −0.18)	−3.02 (−5.73, −0.43)	−3.01 (−4.79, −1.29)	.86
B5 vs D3	−1.74 (−4.39, 0.90)	−2.12 (−4.87, 0.56)	−2.08 (−3.81, −0.39)	.82

(1) Homozygous, (2) heterozygous, (3) recessive, (4) dominant, and (5) allelic genetic models of the following SNPs: (A) CYP1A1 rs4646903:T>C; (B) CYP1A1 rs1048943:A > G; (C) GSTT1 null genotype; (D) GSTM1 null genotype.

CYP1A1 = Cytochrome P450 Family 1 Subfamily A Member 1, GSTM1 = glutathione S-transferase M1, GSTT1 = glutathione S-transferase T1, SNP = single nucleotide polymorphism.

## 4. Discussion

The present analysis included 13 SNPs in 11 genes from 24 studies for pairwise and network meta-analysis. We found that CYP1A1 rs4646903:T>C, CYP1A1 rs1048943:A>G, GSTT1 null genotype, GSTM1 null genotype, and XRCC3 rs861539:C>T were associated with an increased risk of OSF, while MMP2 rs243865:C>T and MMP3 rs3025058:5A>6A correlated with a decreased risk of OSF. Further NMA identified the top 5 genetic models most strongly associated with OSF risk in group 1: the dominant model (ranked 1), homozygous model (rank 2), allelic model (rank 3), recessive model of CYP1A1 rs1048943:A>G (rank 4), and heterozygous/dominant model of CYP1A1 rs4646903:T>C (rank 5). The allelic model of XRCC3 rs861539:C>T and MMP3 rs3025058: 5A>6A ranked first in group 2 and group 3, respectively.

*Network meta-analysis* represents an advanced statistical framework that extends traditional pairwise meta-analysis to enable simultaneous comparisons of multiple interventions or genetic variants within a unified evidence network. Unlike pairwise approaches, NMA integrates both direct and indirect evidence, yielding more precise effect estimates and allowing ranking of relative effects across all included nodes, even when head-to-head comparisons are absent.^[14]^ In genetic association studies, SNP-based network meta-analysis further evaluates multiple genetic models for each polymorphism, identifying the most strongly associated genetic model. In this study, we conducted pairwise meta-analysis to identify SNPs which significantly associated with OSF risk. Then an NMA was conducted to integrate these SNPs to compare their strength of association to OSF risk. The NMA generated 3 independent network groups, including network group 1 (containing various genetic models of CYP1A1 rs4646903:T>C, rs1048943:A>G, GSTT1 null genotype, and GSTM1 null genotype), group 2 (containing various genetic models of XRCC3 rs861539:C>T), and group 3 (containing various genetic models of MMP3 rs3025058: 5A>6A).

*Associations of SNPs in network group 1 to OSF risk*: In network group 1, 4 metabolic gene SNPs were involved, namely CYP1A1 rs4646903:T > C, CYP1A1 rs1048943:A > G, GSTT1 null genotype, and GSTM1 null genotype. CYP1A1 is a phase I metabolic enzyme that oxidizes, reduces, or hydrolyzes xenobiotic and endogenous compounds.^[51]^ Arecoline, an active component of betel nut and a primary etiological agent of OSF, is activated by CYP1A1 to generate reactive oxygen species (ROS), which induce oxidative stress, activate pro-inflammatory/pro-fibrotic pathways, and drive abnormal fibroblasts proliferation and excessive collagen deposition, ultimately leading to OSF.^[51]^ GSTT1 and GSTM1 are key phase II detoxification enzymes that metabolize harmful electrophilic compounds including arecoline, areca nut tannins, and ROS induced by betel quid chewing.^[34]^ CYP1A1 rs4646903:T > C and rs1048943:A > G are 2 common OSF-associated SNPs.^[11]^ Rs4646903, located in a non-coding region, enhances CYP1A1 transcription/expression in response to arecoline, exacerbating tissue damage and fibrosis. Rs1048943:A > G, located in exon 7, alters enzyme structure. Homozygous deletion of GSTT1 or GSTM1 abolishes enzyme expression, leading to deficient detoxification, increased OSF susceptability, and elevated malignant transformation risk. Our pairwise meta-analysis showed that the C allele of rs4646903, the G allele of rs1048943, GSTT1-null genotype, and GSTM1-null genotype were with significant higher risk of OSF (Table 3). To further compare and rank the strength of association between these SNPs and OSF risk, we conducted NMA to integrate these SNPs to compare their strength of association to OSF risk. Our results indicated the top 5 strongly associated genetic models for OSF risk: the dominant, homozygous, allelic, and recessive models of CYP1A1 rs1048943:A>G ranked top 4, while the heterozygous and dominant models of CYP1A1 rs4646903:T>C ranked the 5th. GSTM1 null genotype and GSTT1 null genotype ranked 9 and 12, respectively.

*Associations of SNPs in network group 2 and 3 to OSF risk:* the group 2 and group 3 contains various genetic models of XRCC3 rs861539:C>T and MMP3 rs3025058:5A>6A, respectively. XRCC3, a key component of the homologous recombination DNA repair pathway, cooperates with RAD51 to resolve DNA break ends and ensure accurate repair.^[52]^ The XRCC3 rs861539:C>T polymorphism replaces threonine with methionine at position 241, altering protein structure and impairing binding to partner protein RAD51.^[44]^ MMP3, a members of the matrix metalloproteinase family, degrade extracellular matrix components (e.g., collagen, proteoglycans) to maintain collagen homeostasis.^[53]^ Collagen accumulation is the core pathological event in OSF, leading to excessive extracellular matrix deposition and progressive tissue stiffening. MMP3 rs3025058:5A>6A located in promoter region, directly inhibit MMP3 expression and impair collagen degradation. In group 2, our pairwise meta-analysis revealed that the homozygous, recessive, and allelic models of XRCC3 rs861539:C>T were associated with an elevated risk of OSF. NMA further indicated that the allelic model of XRCC3 rs861539:C>T exhibited the strongest association with OSF risk, followed by the homozygous model and then the recessive model in this group.

Interestingly, we found that the recessive (6A6A vs 6A5A + 5A5A) and allelic (6A vs 5A) models of MMP3 rs3025058:5A>6A were associated with significant lower OSF risk in group 3 (Table 3), with the allelic model exhibiting a stronger effect based on NMA evaluation. The 5A to 6A mutation reduces MMP3 promotor activity, lowering MMP3 expression and inhibited collagen degradation. While this might intuitively predict increased fibrosis risk, the observed protective effect can be explained by the biphasic role of MMP3 in OSF’s pro-fibrotic microenvironment.^[54]^ In OSF, overwhelming collagen synthesis and global MMP inhibition prevail. The 5A allele confers high MMP3 expression; excess MMP3 excessively degrades basement membrane and normal ECM, worsening mucosal injury, inflammation, and fibroblast activation, promoting OSF.^[55]^ In contrast, the 6A allele moderately reduces MMP3, preserving mucosal integrity, limiting inflammation, and counteracting fibrosis, thereby lowering OSF risk.^[54]^ Thus, the 6A variant is protective by restoring ECM balance in OSF’s pathological context.^[31]^

### 4.1. Limitations

First, the evidence base for several SNPs and genetic models is limited, with pooled estimates and NMA comparisons derived from only 2 to 4 primary studies. This small number of included studies constrains statistical power, reduces the precision of effect-size estimates, and introduces uncertainty into the stability of effect estimates and ranking probabilities for different genetic models. Given the total number of studies (n < 10) for these comparisons, leave-one-out sensitivity analysis was not feasible, as single-study omission would further compromise statistical reliability. Instead, we performed a sensitivity analysis by comparing effect estimates from FE and RE models to assess result robustness.^[56]^ For all key associations (e.g., CYP1A1 rs4646903:T>C, CYP1A1 rs1048943:A>G, GSTT1 null genotype, GSTM1 null genotype, MMP2 rs243865:C>T, MMP3 rs3025058:5A>6A, XRCC3 rs861539:C>T), FE and RE models yielded consistent direction of effect and statistical significance, with overlapping 95% confidence intervals. While RE models produced wider confidence intervals (reflecting greater uncertainty from between-study heterogeneity), the core findings remained stable across both modeling approaches. However, conclusions regarding the superiority of 1 model over another, particularly for less-studied loci, still need be interpreted with great caution and viewed as hypothesis-generating rather than definitive.

Second, the lack of subgroup analyses and meta-regression to account for key environmental risk factors. OSF is a multifactorial condition strongly influenced by betel quid chewing, tobacco smoking, and alcohol consumption, exposures that vary widely across populations and may modify genetic effects. Among the 24 included studies, 17 reported chewer proportions in cases and controls (Table S1, Supplemental Digital Content 1), but only 2 studies provided genotype-stratified data, showing higher OSF risk for MMP1 rs1799750 1G/2G and MMP3 rs3025058 6A/6A genotypes in chewers than non-chewers.^[37,40]^ This suggests potential gene-environment interaction, but additional data are required. Formal subgroup analyses and meta-regression were not feasible due to the lack of genotype-stratified exposure data and the Cochrane Handbook’s recommendation of at least 10 studies per covariate for robust meta-regression. Performing such analyses with sparse data would risk false-positive results and data dredging, rather than reliable inference. This constraint means our findings are most generalizable to high-risk Asian populations with heavy betel quid exposure, rather than low-risk or non-Asian groups.

Third, the presence of HWE deviations in control groups and variable methodological quality among included studies. A total of 5 studies showed significant deviation from HWE (*P* < .05), which may indicate potential genotyping errors, population stratification, or selection bias. Additionally, 1 study^[19]^ was rated as low quality, which was excluded from our analysis. Although sensitivity analyses excluding HWE-deviant studies yielded largely consistent effect sizes with the main analysis, these factors may still introduce residual confounding and limit the certainty of estimates.

Fourth, the structure of the NMA network is divided into multiple disconnected subnetworks. The network 1 contains 4 SNPs (CYP1A1 rs4646903, rs1048943, GSTT1 null genotype, and GSTM1 null genotype), while network 2 and 3 only contains various genetic models of XRCC3 rs861539 and MMP3 rs3025058. In a standard NMA framework, isolated SNPs cannot contribute to indirect comparisons, which restricts the possibility of deriving robust comparative rankings across all included SNPs and weakens the interpretability of the results. These limitations mean our findings are most interpretable for SNPs within connected subnetworks, and cross-SNP rankings should be interpreted with caution.

Fifth, we only assessed individual SNPs and not gene-gene interactions among the multiple genes involved in OSF susceptibility. Although network meta-analysis identified several high-ranking predictive genetic models, the combined effects and potential interactions between metabolic genes (e.g., CYP1A1, GSTT1, GSTM1), DNA repair genes (e.g., XRCC3), and matrix remodeling genes (e.g., MMP2, MMP3), DNA repair, and matrix remodeling genes remain unclear due to limited individual-level data and limited genotype information across studies. Therefore, further investigations with larger sample sizes, high-quality data, prospective studies with standardized environmental exposure data, and covering different SNPs to construct a fully connected evidence network are needed.

In summary, our findings indicated that CYP1A1 rs4646903:T>C, CYP1A1 rs1048943:A>G, GSTT1 null genotype, GSTM1 null genotype, XRCC3 rs861539:C>T, MMP2 rs243865:C>T, and MMP3 rs3025058: 5A>6A were significantly associated with OSF risk. Among all evaluated genetic models, the dominant model of CYP1A1 rs1048943:A>G exhibited the strongest association with OSF susceptibility. Future studies with large sample sizes, detailed data on OSF risk factors, and high methodological quality are warranted to further validate the roles of these SNPs in OSF risk.

## Author contributions

**Conceptualization:** Juan Liu, Lele Zhang, Meihua Bao.

**Data curation:** Binsheng He.

**Formal analysis:** Bin Zeng.

**Funding acquisition:** Chunxia Huang, Xiaoshan Huang, Binsheng He.

**Investigation:** Chunxia Huang, Juan Liu, Bin Zeng, Lele Zhang, Zhihang Zhou.

**Methodology:** Zhihang Zhou.

**Project administration:** Juan Liu, Bin Zeng.

**Software:** Juan Liu, Zhihang Zhou.

**Supervision:** Binsheng He, Meihua Bao.

**Visualization:** Lele Zhang.

**Writing – original draft:** Chunxia Huang, Juan Liu, Lele Zhang.

**Writing – review & editing:** Xiaoshan Huang, Binsheng He, Meihua Bao.










