# Supplementary material for: A systematic review and network meta-analysis of single nucleotide polymorphisms associated with oral submucous fibrosis risk
Source: Medicine (Baltimore). 2026 Sep 4;105(36):e50594. doi: 10.1097/MD.0000000000050594 (PMC13549584; doi:10.1097/MD.0000000000050594)
Supplement: Supplementary file 1 [file medi-105-e50594-s001.docx]

**Table S1. The characteristics of included studies**

|  | **Studies** | **Gene/SNP** | **Country** | **Ethnicity** | **Sample size** | | **Males, n (%)** | | **Mean age** | | **betel nut chewing, n(%)** | | **Matched by** | **Genotyping method** | **Quality assessment** |
| --- | --- | --- | --- | --- | --- | --- | --- | --- | --- | --- | --- | --- | --- | --- | --- |
|  |  |  |  |  | **Cases** | **Controls** | **Cases** | **Controls** | **Cases** | **Controls** | **Cases** | **Controls** |  |  |  |
| 1 | Shieh TM,2009 | Lysyl oxidase: rs1800449 | Taiwan | Asian | 83 | 216 | 83(100) | 216(100) | 40.8 ± 12.3 | 49.5 ± 11.1 | 83(100.0) | 216(100.0) | Age, gender | PCR-RFLP | 10 |
| 2 | Ghosh T,2012 | CYP1A1:rs4646903/rs1048943  GSTT1 null genotype  GSTM1 null genotype | India | Asian | 102 | 200 | 77(75.5) | 143(71.5) | 41±12.8 | 39.44±11.1 | Not mention | Not mention | Age, gender | PCR-RFLP | 9 |
| 3 | Mannan A,2024 | GSTT1 null genotype  GSTM1 null genotype | Pakistan | Asian | 108 | 108 | 76(70.4) | 74(68.5) | 25.89±8.7 | 28.8±10.5 | 98(90.7) | 108(100.0) | Age, gender | PCR | 9 |
| 4 | Mukherjee S,2011 | XRCC3: rs rs861539 | India | Asian | 88 | 100 | 48(54.5) | 61(61) | / | / | 83(100.0) | 98(100.0) | Age, gender | PCR-RFLP | 10 |
| 5 | Chaudhuri SR,2013 | CYP1A1:rs4646903/rs1048943 | India | Asian | 75 | 150 | 41(54.7) | 65(43.3) | / | / | 75(100.0) | 150(100.0) | Age, gender | PCR-RFLP | 9 |
| 6 | Mukherjee S,2021 | Lysyl oxidase: rs1800449 | India | Asian | 100 | 100 | 75(75) | 75(75) | / | / | 69(69.0) | 100(100.0) | gender | PCR-RFLP | 10 |
| 7 | Tu HF,2007 | MMP9: rs3918242 | Taiwan | Asian | 73 | 191 | 73(100) | 191(100) | 40.04 ± 11.6 | 47.04 ± 8.9 | 73(100.0) | 191(100.0） | Age, gender | PCR-RFLP | 9 |
| 8 | Chaudhary AK (1),2010 | MMP1: rs1799750 | India | Asian | 412 | 426 | 252(61.2) | 292(69.2) | 42±12.6 | 56±15.6 | 186(45.2) | 188(44.6) | Age, gender | PCR-RFLP | 12 |
| 9 | Madhulatha G,2018 | GSTT1 null genotype  GSTM1 null genotype  MMP9: rs17576 | India | Asian | 25 | 25 | 22(88) | 20(80) | 31.2 | 45.9 | 25(100.0) | 9(36.0) | / | PCR | 7 |
| 10 | Chiu CJ,2002 | Lysyl oxidase: rs1800449  TGFB1: rs1800469 | Taiwan | Asian | 99 | 97 | 99(100) | 97(100) | / | / | 99(100.0) | 97(100.0) | Age, gender | PCR-RFLP | 8 |
| 11 | Tu HF,2008 | p53: rs1042522 | Taiwan | Asian | 66 | 117 | 66(100) | 191(100) | 39.3 ± 10.9 | 46.4 ± 9.4 | 66(100.0) | 117(100.0) | gender | DNA sequencing | 10 |
| 12 | Hallikeri K,2018 | p53: rs1042522 | India | Asian | 30 | 30 | 30(100) | 26(86.7) | / | / | Not mention | Not mention | Age, gender | PCR-RFLP | 6 |
| 13 | Lin YC,2008 | p53: rs1042522 | Taiwan | Asian | 39 | 280 | 39(100) | 133(47.5) | 43.1±9.4 | 52.08±10.2 | 32(82.1) | 31(11.1) | N | PCR-RFLP | 11 |
| 14 | Chaudhary AK,2011 | MMP2: rs243865  MMP9: rs3918242 | India | Asian | 412 | 422 | 252(61.2) | 292(69.2) | 42±12.6 | 56±15.6 | 186(45.2) | 188(44.6) | Age, gender | PCR-RFLP | 11 |
| 15 | Agrawal D,2010 | GSTT1 null genotype  GSTM1 null genotype | India | Asian | 90 | 130 | 71(78.8) | 84(64.6) | 43±15.3 | 40.8±10.3 | Not mention | Not mention | Age, gender | PCR | 9 |
| 16 | Katarkar A,2018 | MMP9: rs3918242/ rs17576 | India | Asian | 189 | 196 | 140(74.07) | 133(67.86) | / | / | 141(74.6) | 0(0.0) | Age, gender | PCR-RFLP | 8 |
| 17 | Yaming P,2016 | CYP1A1: rs1048943 | India | Asian | 95 | 80 | 76(80) | 69(86) | 34.4 | 34.03 | 95(100.0) | 80(100.0) | Age, gender | PCR-RFLP | 8 |
| 18 | Chaudhary AK (2),2010 | MMP3: rs 3025058 | India | Asian | 101 | 126 | 78(77.2) | 105(83.3) | 37.5±12.4 | 31±9.7 | 97(96.0) | 85(67.4) | Age, gender | PCR-RFLP | 10 |
| 19 | Tu HF,2006 | MMP3: rs 3025058 | Taiwan | Asian | 70 | 98 | 70(100) | 98(100) | 38.3±10.6 | 48±9.0 | 70(100.0) | 98(100.0) | gender | DNA sequencing | 8 |
| 20 | Rajendran R,2010 | TGFB1: rs1800469 | India | Asian | 50 | 50 | 36(72) | / | 43.9 | / | 50(100.0) | 50(100.0) | / | DNA sequencing | 7 |
| 21 | Nigam K,2023 | XRCC3: rs rs861539 | India | Asian | 82 | 296 | / | 219(73) | / | 38.02 | Not mention | Not mention | / | PCR-RFLP | 8 |
| 22 | Lin SC (1),2004 | MMP1: rs1799750 | Taiwan | Asian | 58 | 147 | 58(100) | / | 39.1±1.4 | / | 58(100.0) | Not mention | / | DNA sequencing | 11 |
| 23 | Lin SC (2),2004 | MMP2: rs243865 | Taiwan | Asian | 58 | 147 | 58(100) | / | 39.1±1.4 | / | 58(100.0) | Not mention | / | DNA sequencing | 10 |
| 24 | Majumdar PK,2013 | MMP9: rs17576 | India | Asian | 40 | 40 | 24(60) | 25(62.5) | 35.01±12.4 | 35.87±12.2 | Not mention | Not mention |  | PCR-RFLP |  |
