# Supplementary material for: A systematic review and network meta-analysis of single nucleotide polymorphisms associated with oral submucous fibrosis risk
Source: Medicine (Baltimore). 2026 Sep 4;105(36):e50594. doi: 10.1097/MD.0000000000050594 (PMC13549584; doi:10.1097/MD.0000000000050594)
Supplement: Supplementary file 2 [file medi-105-e50594-s002.docx]

**Table S2. Sensitivity analysis compared fixed‑effect and random‑effects data to check pooled effect size and 95% CI consistency**

| **Genetic model** | **Sample size** | **Studies** | **Heterogeneity** | | **Fixed-Effects Model** | **Random-Effects Model** | **Exclusion of HWE violated study^a^** | | |
| --- | --- | --- | --- | --- | --- | --- | --- | --- | --- |
|  | **Case/control** | **N** | **I²(%)** | **P-value** | **OR(95%CI)** | **OR(95%CI)** | **OR(95%CI)/Model** | **I^2^(%)** | **P-value** |
| **LOX rs1800449:G>A** | **282/413** | **3** |  |  |  |  |  |  |  |
| AA vs GG |  |  | 0 | 0.37 | 1.39 [0.68, 2.88] | 1.40 [0.67, 2.91] | 1.10 [0.45, 2.73]/FE | 0 | 0.83 |
| AG vs GG |  |  | 67 | 0.28 | 1.41 [1.00, 2.00] | 1.41 [0.76, 2.61] | 1.18 [0.49, 2.82]/RE | 78 | 0.71 |
| AA vs AG+GG |  |  | 0 | 0.59 | 1.23 [0.60, 2.51] | 1.22 [0.59, 2.53] | 1.00 [0.41, 2.45]/FE | 0 | 0.99 |
| AA+AG vs GG |  |  | 70 | 0.29 | 1.41 [1.01, 1.95] | 1.40 [0.75, 2.58] | 1.15 [0.49, 2.72]/RE | 79 | 0.75 |
| A vs G |  |  | 69 | 0.30 | 1.32 [1.00, 1.74] | 1.30 [0.79, 2.16] | 1.10 [0.56, 2.14]/RE | 75 | 0.79 |
| **CYP1A1 rs4646903:T>C** | **177/350** | **2** |  |  |  |  |  |  |  |
| CC vs TT |  |  | 0 | <0.00001 | 4.35 [2.41, 7.86] | 4.28 [2.36, 7.76] | - | - | - |
| CT vs TT |  |  | 54 | 0.03 | 2.12 [1.35, 3.35] | 2.08 [1.06, 4.08] | - | - | - |
| CC vs CT+TT |  |  | 35 | <0.00001 | 3.27 [2.07, 5.17] | 3.05 [1.59, 5.83] | - | - | - |
| CC+CT vs TT |  |  | 0 | <0.00001 | 2.65 [1.72, 4.09] | 2.67 [1.73, 4.10] | - | - | - |
| C vs T |  |  | 0 | <0.00001 | 2.26 [1.73, 2.97] | 2.26 [1.72, 2.96] | - | - | - |
| **CYP1A1 rs1048943:A>G** | **272/430** | **3** |  |  |  |  |  |  |  |
| GG vs AA |  |  | 83 | 0.0005 | 5.52 [3.72, 8.19] | 5.87 [2.16, 15.97] | 9.67 [5.69, 16.41]/FE | 0 | <0.0001 |
| GA vs AA |  |  | N.A | 0.0003 | 6.19 [2.32, 16.50] | 6.19 [2.32, 16.50] | 6.19 [2.32, 16.50]/N.A | N.A | 0.0003 |
| GG vs GA+AA |  |  | 74 | <0.0001 | 4.13 [2.94, 5.81] | 4.23 [2.14, 8.37] | 5.65 [2.84, 11.25]/RE | 64 | <0.00001 |
| GG+GA vs AA |  |  | 81 | 0.0004 | 5.34 [3.60, 7.93] | 5.55 [2.16, 14.25] | 8.96 [5.26, 15.26]/FE | 0 | <0.00001 |
| G vs A |  |  | 87 | <0.0001 | 4.46 [3.46, 5.74] | 4.52 [2.21, 9.26] | 6.33 [3.81, 10.50]/RE | 62 | <0.00001 |
| **GSTT1 null genotype** | **325/462** | **4** |  |  |  |  |  |  |  |
| Null genotype (-/-) vs Active genotype(+/+ and +/-) |  |  | 0 | <0.00001 | 2.47 [1.69, 3.59] | 2.48 [1.70, 3.61] | - | - | - |
| **GSTM1 null genotype** | **325/462** | **4** |  |  |  |  |  |  |  |
| Null genotype (-/-) vs Active genotype (+/+ and +/-) |  |  | 20 | <0.0001 | 1.81 [1.35, 2.43] | 1.79 [1.28, 2.51] | - | - | - |
| **MMP1 rs1799750:1G>2G** | **470/573** | **2** |  |  |  |  |  |  |  |
| 2G2G vs 1G1G |  |  | 69 | 0.68 | 1.48 [0.96, 2.27] | 1.22 [0.47, 3.18] | - | - | - |
| 2G1G vs 1G1G |  |  | 0 | 0.77 | 0.96 [0.74, 1.25] | 0.96 [0.74, 1.25] | - | - | - |
| 2G2G vs 2G1G+1G1G |  |  | 72 | 0.66 | 1.50 [1.00, 2.25] | 1.24 [0.48, 3.18] | - | - | - |
| 2G2G+2G1G vs 1G1G |  |  | 0 | 0.73 | 1.04 [0.81, 1.34] | 1.04 [0.81, 1.34] | - | - | - |
| 2G vs 1G |  |  | 46 | 0.25 | 1.12 [0.93, 1.35] | 1.06 [0.77, 1.46] | - | - | - |
| **MMP2 rs243865:C>T** | **470/569** | **2** |  |  |  |  |  |  |  |
| TT vs CC |  |  | 12 | 0.35 | 0.74 [0.40, 1.39] | 0.72 [0.28, 1.90] | - | - | - |
| TC vs CC |  |  | 11 | 0.96 | 0.98 [0.51, 1.91] | 0.74 [0.34, 1.58] | - | - | - |
| TT vs TC+CC |  |  | 13 | 0.48 | 0.80 [0.43, 1.49] | 0.77 [0.29, 2.07] | - | - | - |
| TT+TC vs CC |  |  | 0 | 0.03 | 0.73 [0.55, 0.97] | 0.73 [0.55, 0.97] | - | - | - |
| T vs C |  |  | 0 | 0.03 | 0.76 [0.59, 0.97] | 0.76 [0.59, 0.98] | - | - | - |
| **MMP3 rs3025058:5A>6A** | **171/224** | **2** |  |  |  |  |  |  |  |
| 6A6A vs 5A5A |  |  | 0 | 0.35 | 0.49 [0.11, 2.21] | 0.49 [0.11, 2.22] | - | - | - |
| 6A5A vs 5A5A |  |  | 0 | 0.77 | 1.26 [0.26, 6.15] | 1.26 [0.26, 6.18] | - | - | - |
| 6A6A vs 6A5A+5A5A |  |  | 0 | 0.0004 | 0.39 [0.23, 0.66] | 0.39 [0.23, 0.66] | - | - | - |
| 6A6A+6A5A vs 5A5A |  |  | 0 | 0.47 | 0.57 [0.13, 2.61] | 0.58 [0.13, 2.63] | - | - | - |
| 6A vs 5A |  |  | 0 | 0.0005 | 0.44 [0.28, 0.70] | 0.44 [0.28, 0.70] | - | - | - |
| **MMP9 rs3918242:C>T** | **674/809** | **3** |  |  |  |  |  |  |  |
| TT vs CC |  |  | 57 | 0.57 | 1.56 [0.92, 2.67] | 1.35 [0.48, 3.77] | - | - | - |
| TC vs CC |  |  | 0 | 0.57 | 0.93 [0.72, 1.19] | 1.03 [0.76, 1.40] | - | - | - |
| TT vs TC+CC |  |  | 62 | 0.62 | 1.57 [0.93, 2.67] | 1.32 [0.44, 4.00] | - | - | - |
| TT+TC vs CC |  |  | 0 | 0.55 | 1.07 [0.85, 1.36] | 1.08 [0.85, 1.36] | - | - | - |
| T vs C |  |  | 0 | 0.25 | 1.13 [0.92, 1.38] | 1.13 [0.92, 1.38] | - | - | - |
| **MMP9 rs17576:G>A** | **229/236** | **2** |  |  |  |  |  |  |  |
| AA vs GG |  |  | 69 | 0.75 | 1.56 [0.86, 2.83] | 1.23 [0.34, 4.41] | - | - | - |
| AG vs GG |  |  | 51 | 0.75 | 1.29 [0.87, 1.92] | 1.12 [0.54, 2.35] | - | - | - |
| AA vs AG+GG |  |  | 49 | 0.21 | 1.43 [0.81, 2.52] | 1.29 [0.54, 3.11] | - | - | - |
| AA+AG vs GG |  |  | 68 | 0.81 | 1.38 [0.95, 1.99] | 1.11 [0.46, 2.69] | - | - | - |
| G vs A |  |  | 76 | 0.76 | 1.32 [0.99, 1.75] | 1.12 [0.55, 2.28] | - | - | - |
| **XRCC3 rs861539:C>T** | **165/391** | **2** |  |  |  |  |  |  |  |
| TT vs CC |  |  | 43 | <0.0001 | 6.86 [2.63, 17.88] | 6.98 [1.94, 25.12] | - | - | - |
| TC vs CC |  |  | 70 | 0.45 | 1.25 [0.83, 1.89] | 1.35 [0.62, 2.93] | - | - | - |
| TT vs TC+CC |  |  | 62 | 0.02 | 6.14 [2.38, 15.83] | 6.29 [1.35, 29.34] | - | - | - |
| TT+TC vs CC |  |  | 56 | 0.13 | 1.53 [1.04, 2.25] | 1.60 [0.87, 2.93] | - | - | - |
| T vs C |  |  | 5 | 0.0008 | 1.71 [1.25, 2.33] | 1.71 [1.23, 2.36] | - | - | - |
| **TP53 rs1042522:G>C** | **135/371** | **3** |  |  |  |  |  |  |  |
| CC vs GG |  |  | 0 | 0.75 | 1.10 [0.61, 2.01] | 1.10 [0.60, 2.03] | - | - | - |
| GC vs GG |  |  | 0 | 0.44 | 0.83 [0.52, 1.32] | 0.83 [0.52, 1.32] | - | - | - |
| CC vs CG+GG |  |  | 25 | 0.86 | 1.05 [0.61, 1.80] | 1.09 [0.57, 2.10] | - | - | - |
| CC+CG vs GG |  |  | 0 | 0.69 | 0.92 [0.60, 1.42] | 0.92 [0.60, 1.41] | - | - | - |
| C vs G |  |  | 9 | 0.93 | 1.01 [0.75, 1.36] | 0.57 [0.26, 1.28] | - | - | - |
| **TGFB1 rs1800469:T>C** | **215/333** | **2** |  |  |  |  |  |  |  |
| CC vs TT |  |  | N.A | 0.55 | 1.19 [0.68, 2.07] | 1.19 [0.68, 2.07] | - | - | - |
| CT vs TT |  |  | 8 | 0.32 | 0.80 [0.52, 1.23] | 0.82 [0.50, 1.36] | - | - | - |
| CC vs CT+TT |  |  | N.A | 0.13 | 1.44 [0.90, 2.31] | 1.44 [0.90, 2.31] | - | - | - |
| CC+CT vs TT |  |  | 0 | 0.61 | 0.90 [0.60, 1.35] | 0.90 [0.60, 1.35] | - | - | - |
| C vs T |  |  | 0 | 0.61 | 0.:90 [0.60, 1.35] | 1.07 [0.82, 1.40] | - | - | - |

Note: LOX: lysyl oxidase; CYP1A1: Cytochrome P450 Family 1 Subfamily A Member 1; GSTT1: Glutathione S-transferase T1; GSTM1: Glutathione S-transferase M1; MMP-1: Matrix metalloproteinases-1; MMP-2: Matrix metalloproteinases-2; MMP-3: Matrix metalloproteinases-3; MMP-9: Matrix metalloproteinases-9; XRCC3: X‑ray repair cross‑complementing 3; TGF-β1: Transforming growth factor-β1 ; OR: Odds Ratio; CI: confidence intervals; N.A: Not Applicable; FE: fixed-effect model; RE: random-effect model.

^a^, The excluded studies: Mukherjee S, 2021 for LOX rs1800449; Yaming P, 2016 for CYP1A1 rs1048943.
