# Supplementary material for: A systematic review and network meta-analysis of single nucleotide polymorphisms associated with oral submucous fibrosis risk
Source: Medicine (Baltimore). 2026 Sep 4;105(36):e50594. doi: 10.1097/MD.0000000000050594 (PMC13549584; doi:10.1097/MD.0000000000050594)
Supplement: Supplementary file 3 [file medi-105-e50594-s003.docx]

**Table S3 Network of intergene associations in group one**

| A1 | 2.05  (0.34, 12.55) | 0.34  (0.06, 2.26) | 2.43  (0.41, 14.41) | 1.86  (0.31, 10.90) | 3.76  (0.72, 20.91) | 0.18  (0.02, 1.06) | 2.29  (0.43, 12.37) | 3.99  (0.76, 22.91) | 2.82  (0.57, 15.10) | 0.14  (0.02, 0.90) | 0.35  (0.05, 2.35) |
| --- | --- | --- | --- | --- | --- | --- | --- | --- | --- | --- | --- |
| 0.49  (0.08, 2.93) | A2 | 0.17  (0.03, 0.98) | 1.17  (0.21, 6.98) | 0.91  (0.17, 4.96) | 1.84  (0.36, 9.64) | 0.09  (0.01, 0.48) | 1.12  (0.23, 5.66) | 1.97  (0.39, 10.34) | 1.37  (0.27, 7.24) | 0.07  (0.01, 0.43) | 0.17  (0.03, 1.14) |
| 2.92  (0.44, 18.04) | 6.00  (1.02, 33.75) | A3 | 7.03  (1.23, 41.19) | 5.33  (0.97, 30.42) | 10.87  (2.15, 57.33) | 0.53  (0.07, 2.77) | 6.61  (1.37, 33.54) | 11.79  (2.30, 61.36) | 8.09  (1.65, 41.71) | 0.40  (0.06, 2.53) | 1.01  (0.16, 6.50) |
| 0.41  (0.07, 2.42) | 0.86  (0.14, 4.79) | 0.14  (0.02, 0.81) | A4 | 0.77  (0.14, 4.11) | 1.58  (0.32, 8.07) | 0.08  (0.01, 0.39) | 0.96  (0.19, 4.72) | 1.68  (0.34, 8.68) | 1.16  (0.24, 5.74) | 0.06  (0.01, 0.36) | 0.15  (0.02, 0.94) |
| 0.54  (0.09, 3.18) | 1.10  (0.20, 5.96) | 0.19  (0.03, 1.03) | 1.29  (0.24, 7.24) | A5 | 2.04  (0.40, 10.28) | 0.10  (0.01, 0.50) | 1.23  (0.25, 5.90) | 2.16  (0.44, 10.79) | 1.53  (0.31, 7.32) | 0.07  (0.01, 0.43) | 0.19  (0.03, 1.15) |
| 0.27  (0.05, 1.40) | 0.54  (0.10, 2.78) | 0.09  (0.02, 0.46) | 0.63  (0.12, 3.10) | 0.49  (0.10, 2.47) | B1 | 0.05  (0.01, 0.22) | 0.60  (0.15, 2.45) | 1.06  (0.25, 4.49) | 0.74  (0.18, 2.98) | 0.04  (0.01, 0.21) | 0.09  (0.02, 0.51) |
| 5.44  (0.95, 43.72) | 11.04  (2.08, 87.77) | 1.87  (0.36, 14.61) | 12.85  (2.55, 105.29) | 10.00  (2.00, 77.32) | 20.60  (4.52, 137.00) | B2 | 12.32  (2.78, 83.71) | 21.47  (4.98, 151.23) | 15.22  (3.48, 102.30) | 0.74  (0.13, 6.29) | 1.87  (0.32, 15.90) |
| 0.44  (0.08, 2.30) | 0.89  (0.18, 4.44) | 0.15  (0.03, 0.73) | 1.05  (0.21, 5.23) | 0.81  (0.17, 4.02) | 1.66  (0.41, 6.83) | 0.08  (0.01, 0.36) | B3 | 1.76  (0.43, 7.46) | 1.23  (0.30, 4.82) | 0.06  (0.01, 0.34) | 0.15  (0.03, 0.88) |
| 0.25  (0.04, 1.31) | 0.51  (0.10, 2.58) | 0.08  (0.02, 0.43) | 0.59  (0.12, 2.98) | 0.46  (0.09, 2.28) | 0.94  (0.22, 3.99) | 0.05  (0.01, 0.20) | 0.57  (0.13, 2.33) | B4 | 0.70  (0.17, 2.82) | 0.03  (0.01, 0.19) | 0.09  (0.01, 0.48) |
| 0.35  (0.07, 1.76) | 0.73  (0.14, 3.76) | 0.12  (0.02, 0.61) | 0.86  (0.17, 4.24) | 0.65  (0.14, 3.25) | 1.35  (0.34, 5.45) | 0.07  (0.01, 0.29) | 0.81  (0.21, 3.29) | 1.43  (0.35, 5.93) | B5 | 0.05  (0.01, 0.28) | 0.13  (0.02, 0.68) |
| 7.23  (1.11, 51.32) | 14.85  (2.34, 102.18) | 2.53  (0.40, 15.69) | 17.59  (2.74, 120.95) | 13.49  (2.30, 88.18) | 27.28  (4.79, 169.26) | 1.35  (0.16, 7.90) | 16.62  (2.95, 98.31) | 29.14  (5.36, 174.25) | 20.27  (3.62, 119.79) | C3 | 2.53  (0.76, 8.73) |
| 2.87 (0.43, 19.83) | 5.88  (0.88, 38.30) | 0.99  (0.15, 6.28) | 6.85  (1.06, 46.90) | 5.28  (0.87, 33.75) | 10.78  (1.96, 65.58) | 0.54  (0.06, 3.08) | 6.52  (1.14, 39.17) | 11.36  (2.08, 67.21) | 7.98  (1.48, 45.25) | 0.39  (0.11, 1.31) | D3 |
