# Supplementary material for: A systematic review and network meta-analysis of single nucleotide polymorphisms associated with oral submucous fibrosis risk
Source: Medicine (Baltimore). 2026 Sep 4;105(36):e50594. doi: 10.1097/MD.0000000000050594 (PMC13549584; doi:10.1097/MD.0000000000050594)
Supplement: Supplementary file 4 [file medi-105-e50594-s004.docx]

**Table S4 Network of intergene associations in group two**

| E1 | 0.62 (0.22, 1.80) | 2.13 (0.82, 5.69) |
| --- | --- | --- |
| 1.61 (0.55, 4.61) | E3 | 3.41 (1.34, 9.43) |
| 0.47 (0.18, 1.21) | 0.29 (0.11, 0.74) | E5 |
