# Supplementary material for: A systematic review and network meta-analysis of single nucleotide polymorphisms associated with oral submucous fibrosis risk
Source: Medicine (Baltimore). 2026 Sep 4;105(36):e50594. doi: 10.1097/MD.0000000000050594 (PMC13549584; doi:10.1097/MD.0000000000050594)
Supplement: Supplementary file 5 [file medi-105-e50594-s005.docx]

**Table S5 Network of intergene associations in group three**

| F3 | 2.17 (1.03, 4.64) |
| --- | --- |
| 0.46 (0.22, 0.97) | F5 |
